# Supplementary material for: Integrating competency-based assessment into training with peer counsellors providing support at scale to children, adolescents and young adults living with HIV in Zimbabwe: An implementation pilot case study
Source: PLOS Ment Health. 2025 Apr 29;2(4):e0000295. doi: 10.1371/journal.pmen.0000295 (PMC12798172; doi:10.1371/journal.pmen.0000295)
Supplement: Attachment — Submitted filename: Response to Reviewers.docx [file pmen.0000295.s002.docx]

| **Feedback** | **Response** |
| --- | --- |
| ***Journal requirements*** |  |
| Amend financial disclosure | **Thank you for the helpful guidance. This has enabled us to amend our financial disclosure statement to “The authors received no specific funding for this work”** |
| Data storage and availability | **We have anonymised our date to be publicly accessible. We have amended our data statement to, “The data set supporting the conclusions of this article is available on Figshare.com”** |
| Ethics statement | **Thank you for the guidance. We received a waiver from the Medical Research Council of Zimbabwe (MRCZ).**  **institutional review board, to report on these training competency results. We include this information, and explanation, as to why this was appropriate in the methods section. (lines 196-202)** |
| provide an Author Summary. | **We have added a 185-word author summary, and included it between the abstract and introduction as instructed.** |
| ***Reviewer comments*** |  |
| Rev 1: More about program methods:   - 1) It is clear that CATS are 18-24 years of age. It is less clear at what age they become a CATS? How is one chosen to be a CATS? What happens when they exit the program…. when they turn 25? How are CATS compensated? How the # of CATS fluctuates. 2) Do CATS use a training manual or is there a manual of content they are supposed to deliver? There is a mention of how to ensure fidelity…..fidelity to what exactly? To counseling skills? 3) How do clients find CATS? How are CATS paired with clients and what is their goal/objective? Are they paired for a short time, a long time, how many sessions, etc? Do they meet at the clinic, at the client’s household, elsewhere? 4) What exactly does weekly supervision by HCW and monthly by ZDT entail? 5) Is ethics approval needed to report on training-competency results? | **Thank you for highlighting the need to include more detail about the process of becoming a CATS, their role, existing training opportunities and the work that they do in delivering peer counselling to beneficiaries.**  **We have added a text box which concisely explains the details 1) the Zvandiri program, 2) of who the CATS are (how long they are in the program etc), 3) their role/ activities, 4) their training and 5) their supervision. (line 136)**  **We appreciate your query about ethics. We received a waiver from the Medical Research Council Zimbabwe, institutional review board, to report on these training competency results. We include this information, and explanation, as to why this was appropriate in the methods section. (lines 196-202)** |
| Results: 6) The data on integrating EQUIP into training is very compelling, but this was for 16 youth that were identified as needing further training. Can you elaborate on their demographics: how old, what gender, how long had they been working as a CATS? Could this be in comparison to the other CATS that are working, but were not chosen for this particular training?.....i.e. were these 16 younger, a certain gender, less experienced? | **Thank you, we have added the additional description of the participating CATS, including selection and demographics (ages, gender) and length of time as a CATS. (lines 192-193)** |
| Results: 7) These 16 CATS were selected based on their need for ….did the supervisors who selected them comment on their skill, improved or similar post-training (there is data from CATS, but not from the supervisors who suggested they attend the training). That perspective could be helpful. | **Thank you, we appreciate that given the supervisors had both been responsible for their selection to participate in the EQUIP program and continued to work with the CATS post training, that there is considerable value in including their reflections in the results. An explanation of their assessments has been added into the methods and the learning from their reflections is now included within the results. We agree that the integration of their perspective is very helpful and strengthens the paper. (lines 274-276; 369-374)** |
| Discussion 8) There is mention that EQUIP will be incorporated into future Zvandiri trainings. How expensive is this addition? How often do trainings occur given CATS presumably graduate and need to be replaced? How many CATS need to be trained to keep the program quality at scale? Who pays? There is mention of being challenged by constraints in donor funding. Is there room for the MOH in Zimbabwe to support the program? Or what is the path of sustainability? | **We agree that discussing whether and how the EQUIP approach will be incorporated into future Zvandiri trainings is valuable and doing so help us explain why we consider that these findings have a contribution to make to the existing evidence base, as it points to a training pathway that can support sustained quality care at scale. We have included further information explaining how the EQUIP training will be integrated into both the training of new CATS and to supporting the ongoing supervision of existing CATS at a feasible cost within the discussion. We have also added information about how the trainings are funded. (lines 435-442)** |
| 9) Limitations are not discussed. Suggest there are a few limitations in that the N is small, focused on those needing additional support—do all or a few when you consider cost and scale up? if supervisors did not provide input I would consider that a limitation of results, similarly, the perspective of the client (did they feel helped) is missing. | **We have included a paragraph outlining the strengths and limitations of our approach in the final paragraph of the discussion. Within this we emphasise the focus of our enquiry was on the quantitative assessment of an individuals’ counselling skills which can be compared over time and to other individuals. (lines 448-456) The impact of this high-quality counselling on beneficiaries is beyond the scope of what is presented within this paper, but the effect of the CATS’ counselling and support on beneficiaries has been researched previously and we include references to this for the reader to consider in the introduction on lines –128-132.** |
| Reviewer 2:  Reviewer #2: The idea behind this paper is needed and important, unfortunately in it's current state it is unclear whether the manuscript speaks to a specific research question and methods as well as the purpose, scope, and results of the study are not clearly described. | **Thank you, we appreciate that our approach is unusual and that there was a need for us to explain this with greater clarity. We welcome the opportunity to do so.**  **This was not a research study per se, but instead a pragmatic opportunity to present the data and learning from a pilot implementation of the EQUIP competency assessment within an existing effective program- delivering peer counselling. This program is underpinned by an existing strong evidence- base. We conduced a pre and post test of what we describe as an implementation pilot to assess the feasibility and effectiveness of integrating the World Health Organization's EQUIP (Ensuring Quality in Psychological Support) competency-based assessment into existing program training for peer-counsellors, known as CATS (Community adolescent treatment supporters).**  **To assess effectiveness, we compared the baseline and endline levels of the CATS assessed competency, utilising the EQUIP tools and guidance. To assess feasibility, we piloted this approach with existing CATS who their supervisors had identified as in need of further training. The improvement in their scores highlighted that this could work well as a targeted approach. We explain the opportunities for integrating the EQUIP assessment tools into existing training structures in the discussion. To explore whether effectiveness and feasibility was sustained, we followed-up with the CATS and their supervisors seven months after the training and they reported that the training continued to influence their approach and had a sustained positive impact on their counselling practice and self-reported competencies.**  **We have extensively edited the abstract, introduction and methods to more clearly reflect this approach and more effectively explain the remit of our paper. The learning gained from our results is more clearly illustrated through our additional edits within the discussion. We are confident that the contribution of the paper, and the basis of our findings, is clearer and more cogent in our revised manuscript.** |
| - qualitative methods and analysis are not fully described in the methods; how coding was done; or how consent was obtained. | **Thank you, we appreciate that this had not previously been clear. The methods section has been re-written so that the explanation of our methods is clearer. The results we present are based primarily on the numeric assessment scores (baseline, midline and endline) which constitute our pre and post test results.**  **We also include, in line with reviewer 1’s request, the written reflections of the CATS and their supervisors’ seven months later to explore whether the effectiveness of the training in strengthening their competencies was sustained (lines 274-276). This was done through open-text responses to four prompt questions and responses were written brief whatsapp messages. A simple content analysis base don apriori categories was conducted on these brief messages to assess impact. (lines 272-274; 369-274)**  **As explained above, we had an ethics waiver and consent was not sought. This was because the data is derived from program data, collected through the course of this training pilot. CATS consented to participate in the training. The data collected within the training was routine, including the follow-up check-in. We have added a paragraph, Recruitment and Ethics (lines 191-202), explaining this early in the methods, so this is now much clearer to the reader.** |
| - The qualitative findings help support quantitative findings given the small sample size, but as currently written the research questions being answered by the study are not.  Although not suitable for publication with major revisions at this point, we are hopeful the authors might be able to better restructure the paper to highlight the gap in research being filled, the research question, and how it was answered and why. | **Thank you, we hope that the extensive re-writing of our approach and our responses provided above have improved the clarity of our explanation and the overall quality of the paper. The pre and post-test assessment scores of the competencies of the 16 CATS were numeric. We report on these. The follow-up check-in produced open-text written responses from the 16 CATS and their 3 supervisors. We do not frame this as qualitative research. We consider that these are appropriate to assess effectiveness and feasibility of this training approach, and the follow-ups were to inform our understanding of the sustainability of the impact of the training.** |
| - |  |
| ***Additional feedback*** |  |
| - In the Abstract, please include the sample size and a bit about the demographics of the counselor group like mean age. | **Thank you. We have revised the introduction to include more demographic information about the participants. (lines 38-39)** |
| Introduction: |  |
| - Paragraph two would benefit from a bit of clarification or clearer segue into the topic. Please also provide a bit of context into where this is being delivered - MoHCC alone does not make it clear to the reader. More specifics on the length of time and type of support provided (e.g., phone call supervision, in person) would be helpful given the focus of the paper | **Thank you, we have revised the introduction and have added more context, including the text box detailing the CATS intervention. (line 136)** |
| - PAra 3 please include citations to support claims such as ‘skill are not mastered in 1 session’ and the training for professional providers- again context would be important here given training requirements for professionals vary by country | **Thank you, we have reworked this paragraph to more clearly discuss the critical value of training and supervision to support task shifting to lay counsellors, and the importance of a method to assess skill acquisition and competency to support quality care. We have cited relevant references. This information has been added into the introduction (lines 101-106).** |
| - Include context that EQUIP focuses on general counseling skills (i.e., not specific to a treatment) and why you chose a competency based approach to strengthen mentorship and supervision | **Thank you for this recommendation. We have reworked the introduction, methods and discussion sections of the paper, adding context and addressing the issues related to the choice of EQUIP and the competency-based approach to strengthen the counselling skills of the CATS in initial training and ongoing supervision.** |
| - Unclear why and in what way EQUIP being used- to improve selection of providers? To see how well they do after training? Both? | **Thank you for this question. The EQUIP training is being used to improve the quality of the CATS’ counselling skills and to support the maintenance of this over their time as CATS. To ensure that the purpose of this assessment is clear to the reader, we have added this both to the abstract and the penultimate paragraph of the introduction. (lines 140-155)** |
| - The last paragraph is the first time the context of Zimbabwe is mentioned | **Thank you, we have re-written and re-structured the Introduction. The context of Zimbabwe is now mentioned much earlier in the Introduction. (line 124)** |
| Methods |  |
| - More information on “the training” is needed to understand why and how the tool is being used as well as what trainings in the past entailed and why and how enact was included; sections might benefit from benign broken down further by steps such as “Tool Adaption” | **Thank you, we have added a text box that provides more description of the CATS and the standard CATS training. We have also added more description on how the competency assessment was used in pre and post-testing, and how the competencies that were found to need strengthening were integrated into roleplay activities included in the training modules. We have added headings to break down sections. (line 136)** |
| - More information is needed on why adaptations were made and if permission was granted from the equip team - if not then the title or reference may need to be changed to reflect the final tool that was used | **Thank you, we have explained this explicitly within the opening paragraph of the methods section, outlining the collaborative process that was undertaken with WHO to develop a tailored version of the EQUIP approach.** |
| - Who was trained to administer EQUIP? How?" | **The EQUIP tools are available for download on the EQUIP website along with instructional videos, designed to be utilized by mental health professionals with counselling expertise. There is no specific training required for using the competency assessments. The Zvandiri team who are ordinarily responsible for the initial CATS training, led by their clinical psychologist, collaborated with WHO in the adaptation of the tool and in delivering the training.** |
